# Growth-promoting effects of self-selected microbial community on wheat seedlings in saline-alkali soil environments

**DOI:** 10.3389/fbioe.2024.1464195

**Published:** 2024-12-13

**Authors:** Min Li, Wenjie Li, Chunxue Wang, Lei Ji, Kun Han, Jiahui Gong, Siyuan Dong, Hailong Wang, Xueming Zhu, Binghai Du, Kai Liu, Juquan Jiang, Chengqiang Wang

**Affiliations:** ^1^ Shandong Key Laboratory of Agricultural Microbiology, National Engineering Research Center for Efficient Utilization of Soil and Fertilizer Resources, National Key Laboratory of Wheat Improvement, College of Life Sciences, Shandong Agricultural University, Tai’an, China; ^2^ Shandong Provincial Key Laboratory of Applied Microbiology, Ecology Institute, Qilu University of Technology (Shandong Academy of Sciences), Jinan, China; ^3^ State Key Laboratory of Microbial Technology, Institute of Microbial Technology, Shandong University, Qingdao, China; ^4^ State Key Laboratory for Managing Biotic and Chemical Treats to the Quality and Safety of Agro-products, Zhejiang Academy of Agricultural Sciences, Hangzhou, China; ^5^ Department of Microbiology and Biotechnology, College of Life Sciences, Northeast Agricultural University, Harbin, China

**Keywords:** saline-alkali soil environment, the rhizosphere microenvironment, metagenome, wheat, plant growth-promoting rhizobacteria

## Abstract

Saline-alkali land is a type of soil environment that causes poor crop growth and low yields. Its management and utilization are, therefore of great significance for increasing arable land resources, ensuring food security, and enhancing agricultural production capacity. The application of plant growth-promoting rhizobacteria (PGPR) is an effective way to promote the establishment of symbiotic relationships between plants and the rhizosphere microenvironment, plant growth and development, and plant resistance to saline-alkali stress. In this study, multiple saline-alkali-resistant bacteria were screened from a saline-alkali land environment and some of them were found to have significantly promotive effects on the growth of wheat seedlings under saline-alkali stress. Using these PGPR, a compound microbial community was selectively obtained from the root-zone soil environment of wheat seedlings, and the metagenomic sequencing analysis of wheat root-zone soil microbiomes was performed. As a result, a compound microbial agent with a *Kocuria dechangensis* 5–33:*Rossellomorea aquimaris* S-3:*Bacillus subtilis* BJYX:*Bacillus velezensis* G51-1 ratio of 275:63:5:1 was obtained through the self-selection of wheat seedlings. The synthetic compound microbial agent significantly improved the growth of wheat seedlings in saline-alkali soil, as the physiological plant height, aboveground and underground fresh weights, and aboveground and underground dry weights of 21-day-old wheat seedlings were increased by 27.39% (*p* < 0.01), 147.33% (*p* < 0.01), 282.98% (*p* < 0.01), 194.86% (*p* < 0.01), and 218.60% (*p* < 0.01), respectively. The promoting effect of this compound microbial agent was also greater than that of each strain on the growth of wheat seedlings. This microbial agent could also regulate some enzyme activities of wheat seedlings and the saline-alkali soil, thereby, promoting the growth of these seedlings. In this study, we analyze an efficient microbial agent and the theoretical basis for promoting the growth of wheat seedlings under saline-alkali stress, thereby, suggesting an important solution for the management and utilization of saline-alkali land.

## Introduction

Increasing the crop cultivation area and yield is an important measure for ensuring the economic development of human society, and the soil environment and quality can seriously affect crop yields and quality (Zhang S. et al., 2022; Song et al., 2022). With the change in climate and the effect of human activities, many soils have become saline-alkaline, leading to a continuous reduction in arable land and a decrease in the utilization of agricultural resources (Li et al., 2022; Ali et al., 2022). Land salinization can lead to a soil nutrient imbalance through water and salt stresses, thereby hindering plant growth (Jin et al., 2019). Therefore, the management and efficient use of saline-alkali soil is of great significance for ensuring food security, increasing arable land resources, and enhancing agricultural productivity (Kumawat et al., 2023; Yuan et al., 2021).

The use of saline-alkali-resistant microorganisms to improve these soils is an important strategy for solving soil salinization (Ren et al., 2023; Xiao et al., 2022). Research has shown that the maintenance of a low Na^+^ concentration and high K^+^ concentration in the cytoplasm and cytoplasmic solutes is necessary for the growth and development of plants under salt stress (Derbyshire et al., 2022). Salt-tolerant microorganisms can reduce Na^+^ intake by crops and alleviate the impact of salt stress on crop cells (Sahu et al., 2021). Applying microbial agents comprising salt-tolerant microorganisms can also increase the soil nutrient content, facilitate the breakdown of substances that are difficult for plants to utilize and the degradation of harmful substances, increase beneficial microorganism levels in saline-alkali soil, improve soil respiration, reduce soil salinity, and thereby increase crop growth and yields (Shi et al., 2022). Accordingly, the use of plant growth-promoting rhizobacteria (PGPR) to alleviate saline-alkali stress on plants is considered an important sustainable agriculture measure (Mukherjee et al., 2019). PGPR are beneficial bacteria that establish symbiotic relationships with plants in the rhizosphere microenvironment, and they can promote the growth and development of plants through various mechanisms, increasing the resistance of plants to saline-alkali stress and other adverse conditions (Garcia-Lemos et al., 2020; Wang et al., 2020). Salt-tolerant bacteria isolated from the rhizosphere of halophytes can maintain ion homeostasis in plants and promote plant growth under saline-alkali stress conditions by producing substances such as indole-3-acetic acid, ACC deaminase, and extracellular polysaccharides (Sharma et al., 2016; Mukherjee et al., 2019). Further, the use of salt-tolerant PGPR is an effective method that has been successfully applied to a variety of crops to improve their growth and tolerance under saline-alkali stress conditions (Sharma et al., 2016). Considering various saline-alkali areas and different crops, the development of distinct, saline-alkali-tolerant, and growth-promoting agents has great application value, necessitating their further development (Ji et al., 2021).

Wheat (*Triticum aestivum* L.) is a staple food and contributes to approximately 35% of global food grains; moreover, the use of saline-alkali land to grow wheat has great value (Arshad et al., 2024). Some salt-tolerant PGPR have been applied to wheat to improve their growth and tolerance under saline-alkali stress conditions. For example, *Bacillus velezensis* JC-K can function in saline-alkali soil, effectively increase wheat biomass accumulation and osmotic regulatory capacity, and significantly improve the absorption of Na^+^ and K^+^ in wheat (Elliott et al., 2021). *Bacillus methylotrophicus* M4-1 can affect the physiological and biochemical traits of wheat and protect winter wheat from the harmful effects of soil salinity and alkalinity (Ji et al., 2020). Moreover, the co-application of *Paenibacillus sabinae* (potassium-solubilizing bacteria) and *Leptolyngbya* sp. RBD05 (cyanobacteria) improves saline-alkali stress tolerance in wheat (Duan et al., 2023). Therefore, salt-tolerant and beneficial PGPR in the wheat rhizosphere have important applications and value for the future biological restoration of saline-alkali land to improve wheat yields (Hou et al., 2021). However, at present, the development of compound microbial agents for wheat has often been based on artificial compatibility, with a poor affinity for environments such as wheat-growing and saline-alkali land. It is, therefore, very important to construct a microbial agent with high affinity and high colonization capacity for wheat planted in saline-alkali land. As such, new technical methods are also needed to develop novel, specific, and high-affinity compound microbial agents for wheat planted in saline-alkali land.

The focus of this study was the development of a new type of specific compound microbial agent with a high affinity for wheat seedlings in saline-alkali land. An *in situ* self-selection strategy, based on the rhizosphere microenvironment of wheat seedlings, was used to develop the new microbial agent, and its protective effects in saline-alkali environments and growth-promoting characteristics were also tested. This study also provides an important basis for further research on the mechanism underlying microorganism and crop interactions and the efficient utilization of saline-alkali soil.

## Materials and methods

### Wheat variety and soil materials

A hybrid No. 22 wheat variety was selected, and it was bred by the Crop Research Institute of Shandong Academy of Agricultural Sciences. Normal field soil (salt content of 0.06%) from Taian, China, and raw saline-alkali soil (salt content of approximately 0.8%, organic matter content of approximately 0.65%, and pH 7.9) from Dongying, China, were selected for this study.

### Screening and identification of rhizobacteria with saline-alkali resistance

Ten grams of raw saline-alkali soil was added to 100 mL of deionized water and shaken at 37°C and 180 rpm for 30 min; then, 100 µL of the diluted liquid was spread onto LB culture medium containing 8% NaCl for culture at 37°C for 48–72 h. Single colonies with different sizes and shapes were isolated and purified via three-zone streaking. The purified strains were cultured in an LB liquid medium, and then, 50 µL of the corresponding liquid obtained from the initial screen was spread onto LB solid media with different concentrations of NaCl, ranging from 0% to 17%, or with different acidic and alkaline pH values for incubation and observations of the growth capacity. Gram staining of bacterial cells was conducted to observe the cell morphology, and bacterial microbiochemical identification tubes (Haibo Biotechnology Co., Ltd., China) were used to determine some physiological and biochemical characteristics of the strains (Zhao et al., 2022). The 16S rDNA (amplified using 27F/1492R primers) and *gyrB* gene (amplified using the primers gyrB-F “GAT​CAA​CTA​ACA​GCA​AAG​GCC​TTC​ACC​A” and gyrB-R “AGA​CCT​TTA​TAA​CGC​TGG​AGG​CCG​GGC​T”) of bacteria were obtained for sequencing (Shengong Biotechnology Co., Ltd., China) (Ye et al., 2022). Combining the morphological characteristics of cells, physiological and biochemical indicators, *gyrB* gene sequence comparison, and phylogenetic tree analysis of 16S rDNA sequences (Constructed using MEGA 11.0), the bacterial species were identified (Zhao et al., 2022; Rahma et al., 2020).

### Wheat seedling pot experiments using simulated saline-alkali soil or saline-alkali soil from raw saline-alkali land

The normal field soil was first sieved through an 18-mesh sieve and then mixed with vermiculite at a volume ratio of 2:1, and the resulting soil was used for the pot experiment comprising simulated saline-alkali soil. For the pot experiment using raw saline-alkali soil, the raw saline-alkali soil from Dongying was mixed with vermiculite at a 1:1 volume ratio and then mixed with 2% chicken organic manure. For the wheat pot experiment, 0.5 kg of the soil mixture was added to each pot.

Under dark cultivation conditions for 8 h, wheat seeds were soaked in sterile water until budding, and 3–5 wheat seeds with the same budding characteristics were selected and planted in a pot. After covering them with a thin coat of soil, as in the pot experiment using simulated saline-alkali soil, 100 mL of salt water with a mixture of 0.655 mM NaCl, 5.85 mM Na_2_SO_4_, 5.85 mM NaHCO_3_, and 0.65 mM Na_2_CO_3_ (pH = 8.3) was added, and the same amount of water was added in the control group. The wheat seedlings were cultivated at 20°C under 40,000 lx light and 0 lx darkness conditions. After 48 h of cultivation, wheat seedlings with small growth differences were retained in the pots. Strains were activated twice using an LB liquid medium. Each pot was watered with 100 mL of a bacterial suspension with approximately 10^6^ CFU mL^−1^ (single strain or mixed strains), and the same amount of water was added to the control group. During the wheat seedling growth process, 30 mL of water was used to water each pot every 2 days, and the physiological plant height was measured every 7 days.

During the pot experiment using simulated saline-alkali soil, the data of the conventional agronomic traits of wheat seedlings were recorded after growth for 14 days, as reported (Wang et al., 2024). During the pot experiment using raw saline-alkali soil, the data of conventional agronomic traits, dry and fresh weights, leaf and root enzyme activities, root system, soil enzymatic activities, and malondialdehyde (MDA) concentrations in wheat seedlings were recorded after growth for 21 days as reported (Wang et al., 2024). The nitroblue tetrazolium photochemical reduction method was used to determine superoxide dismutase (SOD) enzyme activity (Begum et al., 2022), the pyrogallol autoxidation method was used to determine peroxidase activity (Wang J. et al., 2022), catalase (CAT) activity was measured based on the reaction comprising the catalase-mediated degradation of hydrogen peroxide (Li et al., 2019), and the MDA concentration was determined based on its reaction with thiobarbituric acid (Pervaiz et al., 2021). Different enzyme activity assay kits (Suzhou Grice Biotechnology Co., Ltd., China) were used to measure the activities of neutral phosphatase, alkaline phosphatase, acid phosphatase, catalase, urease, and sucrase in wheat rhizosphere soil or non-rhizosphere soil.

Furthermore, the effects of the strains on the germination of the wheat seeds were also tested under saline-alkali conditions with a mixture of 0.655 mM NaCl, 5.85 mM Na_2_SO_4_, 5.85 mM NaHCO_3_, and 0.65 mM Na_2_CO_3_ (pH = 8.3). The procedures were performed as reported (Wang K. et al., 2022).

### Self-selected experiment of the microbial community of wheat seedlings in a rhizosphere microenvironment

The raw saline-alkali soil was mixed with vermiculite at a 1:1 volume ratio and then sterilized. Then, 0.5 kg of the soil mixture was added to each pot. The wheat seedling sowing and cultivation methods were the same as those mentioned previously herein. Strains with good growth-promoting effects were selected for the wheat seedling self-selection experiment. Each strain was cultivated twice in an LB liquid medium. Then, equal amounts of bacteria were mixed at a 1:1 ratio. The bacterial suspension was diluted 100 times and then applied to the potted wheat seedlings. After 21 days, root zone soil samples were collected for metagenomic sequencing to determine the colonization ratio of different bacteria.

### Metagenomic sequencing of rhizosphere soils

The complete DNA of the root zone soil was extracted using the OMEGA Mag-Bind soil DNA Kit (Omega Bio-Tek, United States) and then sequenced at Shanghai Personalbio Technology Co., Ltd. (Personalbio, China) for the metagenomic analysis. Three biological samples (SSP_1, SSP_2, and SSP_3) were used. The different insert fragment libraries were constructed using the whole-genome shotgun strategy. Then, next-generation sequencing technology, based on the Illumina NovaSeq sequencing platform, and third-generation single-molecule sequencing technology, based on the Oxford Nanopore ONT sequencing platform, were used to sequence these libraries (Xiao et al., 2021). The sequencing adapters were removed from reads using Cutadapt (v1.2.1) (Martin, 2011). The original metagenomic sequencing data can contain some low-quality sequences, the presence of which could interfere with subsequent analyses. To ensure the reliability of the subsequent analysis, fastp (v0.23.2) software was used to screen and filter the raw data obtained from sequencing (Chen et al., 2018). Once quality-filtered reads were obtained, taxonomical classifications of metagenomics sequencing reads from each sample were performed using the method Kaiju (v1.9.0) against an NCBI-nt-derived database (Menzel et al., 2016). A tool specifically designed to predict genes in metagenomic sequences was used for gene prediction (Zhang X. et al., 2022). Megahit (v1.1.2) was used for assembly of reads in each sample using the meta-large presetted parameters. In MEGAN (Metagenome Analyzer, http://ab.inf.uni-tuebingen.de/software/megan5/) software, the lowest common ancestor algorithm was used for the species classification annotation information of the target sequence (Huson et al., 2016). R software was used to generate bar charts of the compositions of dominant species at various taxonomic levels in each sample (Deng et al., 2020).

## Results

### Screening and identification of saline-alkali-tolerant microorganisms

**FIGURE 1:**
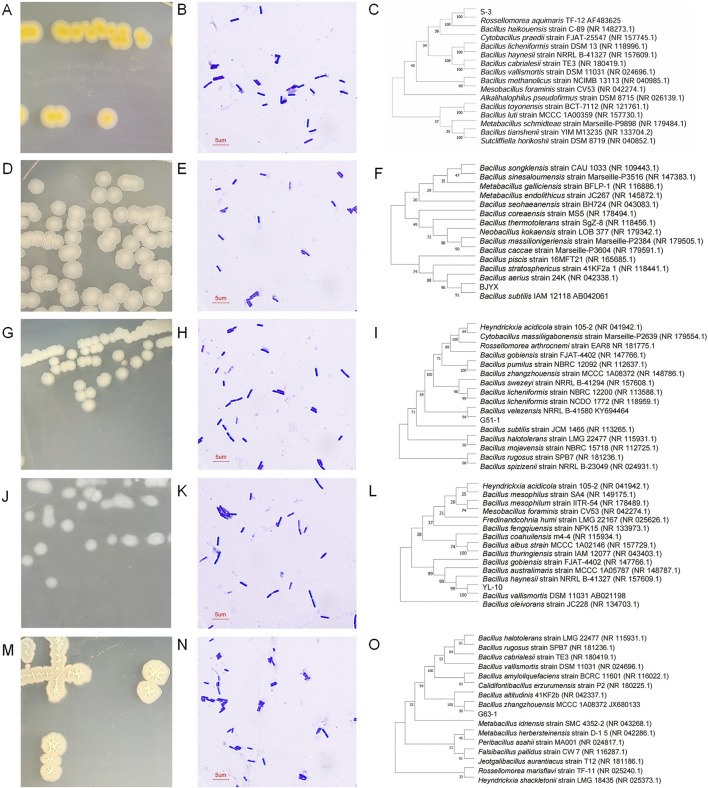
Colony, cell morphology, and phylogenetic tree analysis of selected strains. Strains were activated using LB media. The phylogenetic tree analysis of 16S rDNA sequences of strains was performed using MEGA 11.0. Colony **(A)**, cell morphology **(B)**, and phylogenetic tree **(C)** analysis of strain S-3; colony **(D)**, cell morphology **(E)**, and phylogenetic tree **(F)** analysis of strain BJYX; colony **(G)**, cell morphology **(H)**, and phylogenetic tree **(I)** analysis of strain G51-1; colony **(J)**, cell morphology**(K)**, and phylogenetic tree **(L)** analysis of strain YL-10; colony **(M)**, cell morphology **(N)**, and phylogenetic tree **(O)** analysis of strain G63-1.

In total, 151 strains were first isolated from the raw saline-alkali soil, and then, five highly saline-alkali-tolerant strains, namely, S-3, BJYX, G51-1, YL-10, and G63-1, were finally screened. The NaCl tolerance limits of strains S-3, BJYX, G51-1, YL-10, and G63-1 on plates were 16, 11, 12, 12, and 12%, respectively, and their alkali tolerance limits were pH 14, 13, 10, 8, and 11, respectively. Strains S-3, BJYX, G51-1, YL-10, and G63-1 were all found to be Gram-positive bacteria. The colonies of strain S-3 were milky yellow, opaque, and circular in shape; the colonies of strain BJYX were milky white, semi-transparent, and circular in shape; the colonies of strain G51-1 were beige, opaque, and irregular in shape; the colonies of strain YL-10 were gray-white, opaque, and circular in shape; and the colonies of strain G63-1 were milky white, semi-transparent, and irregular in shape, and they were all able to produce spores (Figure 1). The 16S rDNA gene sequences of the strains were analyzed through the generation of phylogenetic trees to search for closely related species (Figure 1), and the results were further verified based on the *gyrB* gene sequence of each strain. Finally, it was determined that strain S-3 is *Rossellomorea aquimaris*, strain BJYX is *Bacillus subtilis*, strain G51-1 is *Bacillus velezensis*, strain YL-10 is *Bacillus vallismortis*, and strain G63-1 is *Bacillus zhangzhouensis*.

### Effects of strains on the growth of wheat seedlings using simulated saline-alkali soil

**FIGURE 2:**
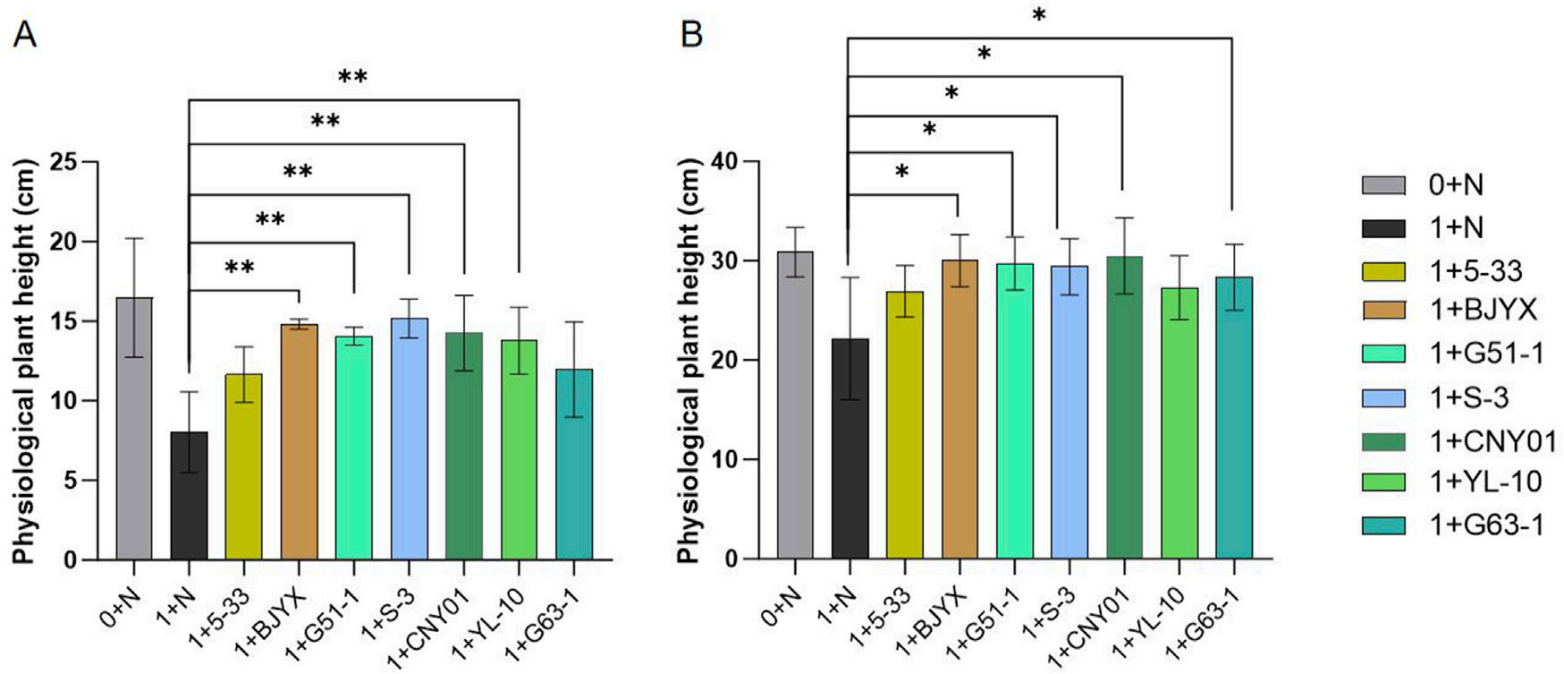
Effects of strains on physiological plant heights of wheat seedlings using simulated saline-alkali soil. “0+” represents the normal field soil, “1+” represents the simulated saline-alkali soil. At the early stage of wheat germination, various microbial agents were applied, and 30 mL of water was applied every 2 days. The plant heights were measured at 7 days **(A)** and 14 days **(B)** after wheat growth. Each group was subject to 3–5 biological treatments. The averages of the data are presented. Error bars represent the standard deviations of the averages. *: *p* < 0.05, significant; **: *p* < 0.01, highly significant.

All newly screened saline-alkali-tolerant PGPR and the previously preserved strains *Kocuria dechangensis* 5–33 (Wang et al., 2015) and *Bacillus atrophaeus* CNY01 (Sun et al., 2024) were tested for their capacity to affect wheat growth using pot experiments under simulated saline-alkali conditions of a moderate environment. After 7 days (Figure 2A) and 14 days (Figure 2B) of wheat seedling growth, the addition of strains 5-33, BJYX, G51-1, S-3, CNY01, YL-10, and G63-1 had obvious growth-promoting effects on wheat seedlings, as follows: for 7 days growth, physiological plant heights were increased by 44.95%, 84.44% (*p* < 0.01), 74.89% (*p* < 0.01), 88.81% (*p* < 0.01), 77.29% (*p* < 0.01), 71.49% (*p* < 0.01), and 48.94% (*p* < 0.01), respectively (Figure 2A); for 14 days growth, the physiological plant heights were increased by 21.55%, 35.36% (*p* < 0.05), 34.07% (*p* < 0.05), 32.66% (*p* < 0.05), 37.54% (*p* < 0.05), 23.18%, and 27.89% (*p* < 0.05), respectively, compared with those in the control group (Figure 2B). All strains promoted the growth of wheat seedlings in the simulated saline-alkali soil, and almost all of them resulted in a wheat growth trend similar to that in normal soil.

### Self-selected microbial community based on wheat rhizosphere

Equal amounts of strains S-3, BJYX, G51-1, YL-10, G63-1, 5-33, and CNY01 were added to the rhizosphere microenvironment of wheat seedlings. After cultivation for 21 days, the root-zone soil of wheat seedlings was collected for metagenomic sequencing to determine the colonization capacity and ratio of the different bacteria added. During the metagenomic sequencing process, the numbers of raw reads generated from three samples were 101,824,340, 102,932,646, and 93,404,390, respectively. Meanwhile, the numbers of quality-filtered reads generated from three samples were 99,170,304, 99,887,882, and 91,054,674, respectively. The minimum read length before sticking paired-end reads was 150 bp. After the concatenation was complete, contigs with a length of not less than 300 bp were retained. For the metagenomic sequencing data, the proportion of valid sequences was greater than 96%, the proportion of valid sequence base numbers was also greater than 96%, and the slopes of the rarefaction curves became smaller and tended to flatten at last (Supplementary Figure S1), indicating that samples could be further analyzed.

### Microbial compositions in the rhizosphere soil at the phylum level

**FIGURE 3:**
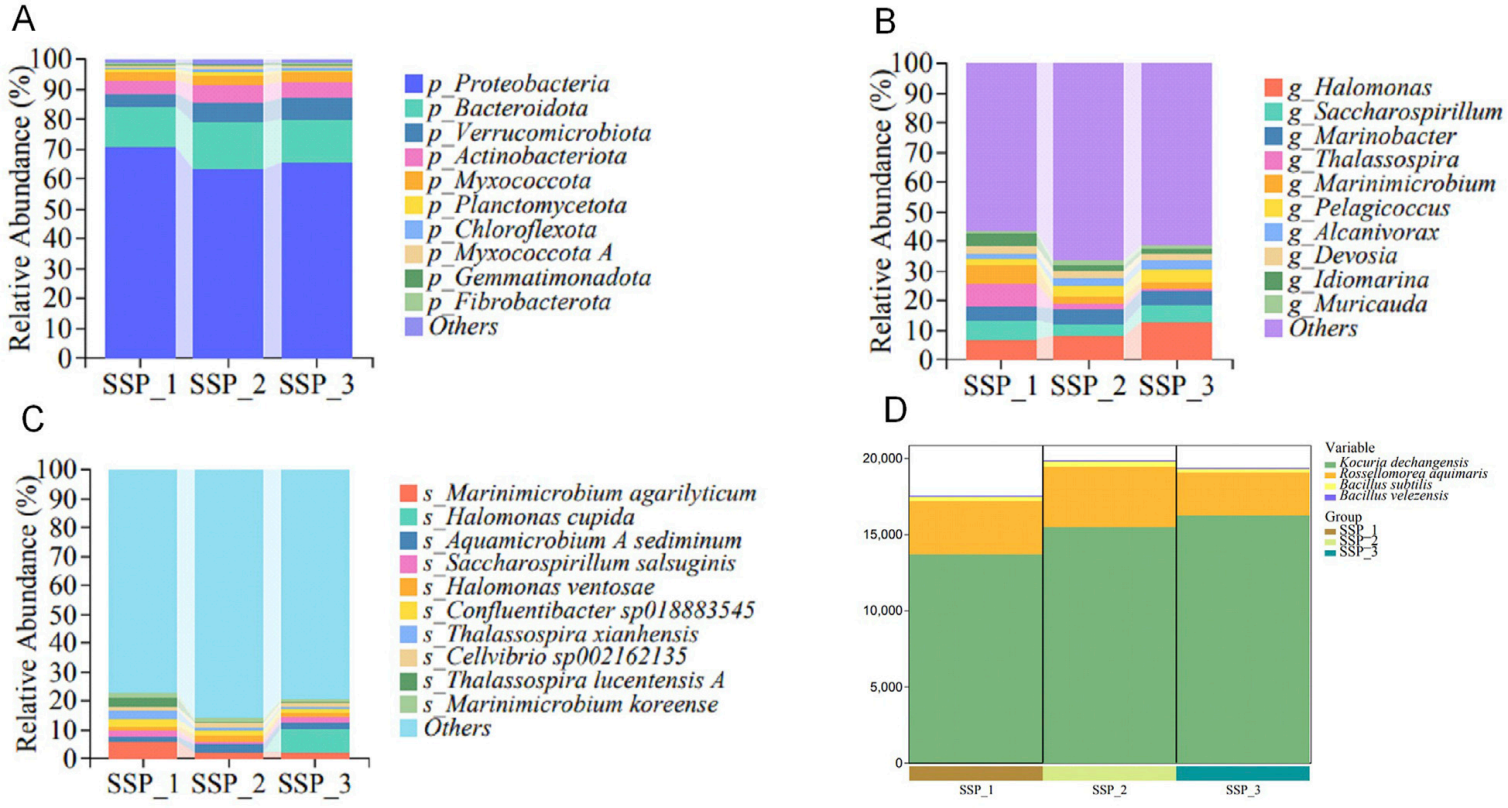
Analysis of taxonomic compositions at the phylum **(A)**, genus **(B)**, and species **(C)** levels, and the bacterial numbers of the added species obtained via metagenomic sequencing **(D)**. The relative abundances (top 10) of different microbial community structures at the phylum, genus, and species levels with the three treatments (SSP_1, SSP_2, and SSP_3) are presented.

In terms of the community composition of the rhizosphere soil of wheat seedlings, the top 10 phyla of bacteria with the highest proportions were determined, and the levels ranged from high to low and included Proteobacteria, Bacteroidetes, Verrucomicrobia, Actinobacteria, Myxococcota, Planctomycetota, Chloroflexota, Myxococcota A, Gemmatimonadetes, and Fibrobacterota (Figure 3A). Among them, Proteobacteria was the dominant phylum in the rhizosphere soil of wheat in all treated samples, with an average relative abundance of 66.5%. The relative abundances of the other phyla Bacteroidetes, Verrucomicrobia, Actinobacteria, Myxococcota, Planctomycetota, Chloroflexota, Myxococcota A, Gemmatimonadetes, and Fibrobacterota were 14.70, 5.75, 5.20, 3.14, 0.97, 0.97, 0.64, 0.58, and 0.31%, respectively.

### Microbial compositions in the rhizosphere soil at the genus level

In terms of the community composition of the rhizosphere soil of wheat seedlings, the top 10 genera with the highest proportions were determined, and the levels ranged from high to low, including *Halomonas*, *Saccharospirillum*, *Marinobacter*, *Thalassospira*, *Marinimicrobium*, *Pelagicoccus*, *Alcanivorax*, *Devosia*, *Idiomarina*, and *Muricauda* (Figure 3B). Among them, *Halomonas* was the dominant genus in the wheat rhizosphere soil of all treatment samples, with an average relative abundance of 9.36%, whereas the average proportions of the other genera *Saccharospirillum*, *Marinobacter*, *Thalassospira*, *Marinimicrobium*, *Pelagicoccus*, *Alcanivorax*, *Devosia*, *Idiomarina*, and *Muricauda* were 5.05, 4.62, 3.79, 3.36, 3.26, 2.40, 2.38, 2.29, and 1.75%, respectively.

### Microbial compositions of the rhizosphere soil at the species level

In terms of the community composition of the rhizosphere soil of wheat, the top 10 species with the highest proportions were determined, and the levels ranged from high to low, including *M. agarilyticum*, *H. cupida*, *A. sediminum*, *S. salsuginis*, *H. ventosae*, *Confluentibacter* sp018883545, *Thalassospira xianhensis*, *Cellvibrio* sp002162135, *Thalassospira lucentensis A*, and *M. koreense* (Figure 3C). Among them, *Marinimicrobium agarilyticum* was the dominant species in the rhizosphere soil of wheat seedlings in all treatment samples, with an average relative abundance of 3.28% in the three parallel samples, whereas the average proportions of the other species *Halomonas cupida*, *Aquamicrobium sediminum*, *Saccharospirillum salsuginis*, *Halomonas ventosae*, *Confluentibacter* sp018883545, *T*. *xianhensis*, *Cellvibrio* sp002162135, *T*. *lucentensis A*, and *Marinimicrobium koreense* were 2.79, 1.98, 1.92, 1.62, 1.60, 1.58, 1.49, 1.22, and 1.20%, respectively.

Bacterial numbers for the added species were also determined (Figure 3D). The species *K. dechangensis* 5–33, *R. aquimaris* S-3, *B. subtilis* BJYX, and *B. velezensis* G51-1 ultimately colonized the rhizosphere soil of wheat seedlings at a ratio of 275:63:5:1. This suggests that these four species have a high affinity for the wheat rhizosphere microenvironment.

### Effects of the selected microbial community on wheat seedlings using raw saline-alkali soil

To verify that the self-selected microflora in the wheat rhizosphere microenvironment has better saline-alkali resistance and growth-promoting effects on wheat seedlings, pot experiments using raw saline-alkali soil were conducted, with a control group and six treatment groups. The six treatment groups comprised four single-strain treatments (strains 5-33, S-3, BJYX, and G51-1), a mixed treatment with an equal proportion of the four selected strains (MBE group), and a mixed treatment with a self-selected proportion (selected by wheat seedling) of the four selected strains (MB group).

**FIGURE 4:**
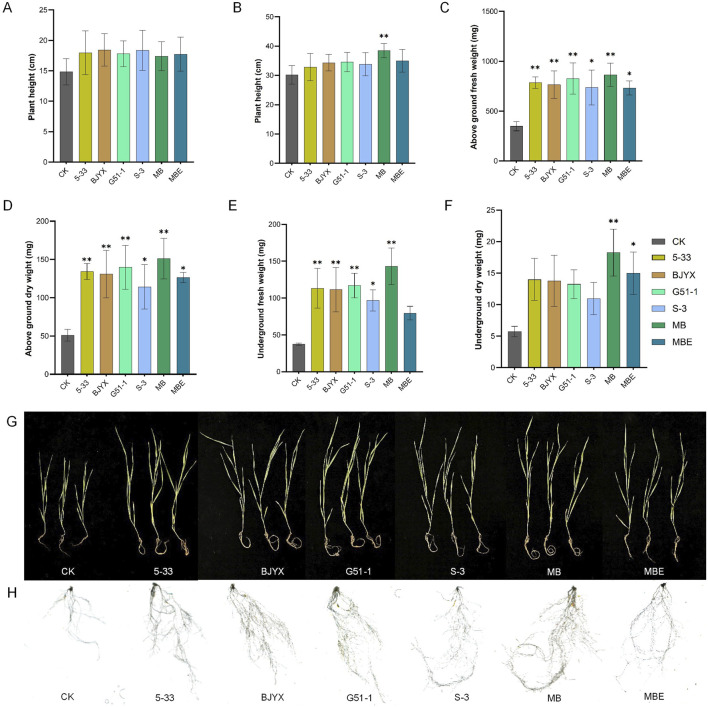
Agronomic traits of wheat seedlings under different treatment conditions. When the wheat sprouted, the strain inoculants were added, and then, the plants were watered every 2 days with 30 mL of water. The heights of the wheat seedlings after 7 days **(A)** and 21 days **(B)** were recorded. The fresh weights of the aboveground parts **(C)** and the underground parts **(D)**, the dry weights of the aboveground parts **(E)** and the underground parts **(F)** after drying for 48 h, the complete wheat seedlings **(G)**, and the roots of the wheat seedlings **(H)** after 21 days of growth are displayed. “MB” represents the self-selected mixed bacteria, and “MBE” represents the mixed bacteria of the selected strains based on an equal proportion. Each group was subjected to 3–5 biological treatments. The averages of the data are presented. Error bars represent the standard deviations of the averages. *: *p* < 0.05, significant; **: *p* < 0.01, highly significant.

The physiological plant heights of wheat seedlings cultivated for 7 and 21 days after the strains were added are shown in Figure 4A. For all six treatment groups, the physiological plant heights were all greater than those of the control group. Compared with those of the control group, the physiological plant heights of the 5-33, BJYX, G51-1, S-3, MB, and MBE treatment groups were increased by 21.01, 24.23, 19.89, 23.70, 17.28, and 19.45%, respectively, at 7 days (Figure 4A). After the wheat seedlings were cultivated for 21 days, compared to those in the control group, the physiological heights in the 5-33, BJYX, G51-1, S-3, MB, and MBE treatment groups were increased by 8.86, 13.72, 14.49, 11.95, 27.39 (*p* < 0.01), and 15.92%, respectively (Figure 4B); the aboveground fresh weights of the 5-33, BJYX, G51-1, S-3, MB, and MBE treatment groups were increased by 125.06% (*p* < 0.01), 119.12% (*p* < 0.01), 136.76% (*p* < 0.01), 110.82% (*p* < 0.05), 147.33% (*p* < 0.01), and 109.91% (*p* < 0.05), respectively (Figure 4C); the aboveground dry weights of the 5-33, BJYX, G51-1, S-3, MB, and MBE treatment groups were increased by 162.09% (*p* < 0.01), 155.52% (*p* < 0.01), 173.02% (*p* < 0.01), 123.01% (*p* < 0.05), 194.86% (*p* < 0.01), and 146.61%(*p* < 0.05), respectively (Figure 4D); the underground fresh weights of the 5-33, BJYX, G51-1, S-3, MB, and MBE treatment groups were increased by 203.12% (*p* < 0.01), 198.31% (*p* < 0.01), 213.19% (*p* < 0.01), 159.27% (*p* < 0.05), 282.98% (*p* < 0.01), and 112.92%, respectively (Figure 4E); and the underground dry weights of the 5-33, BJYX, G51-1, S-3, MB, and MBE treatment groups were increased by 144.77%, 140.69%, 131.39%, 91.28%, 218.60% (*p* < 0.01), and 161.63% (*p* < 0.05), respectively (Figure 4F).

An observation of wheat seedlings with different treatments (Figure 4G) and root scanning (Figure 4H) revealed that wheat seedlings treated with the inoculants of the strains showed better growth compared to the control, and various root indicators were increased by different degrees compared to those in the control group (Table 1). Especially in the MB treatment group, compared to those in the control group, all agronomic trait data were optimal. Therefore, the *in situ* self-selected strain treatment had a better promoting effect on wheat in response to saline-alkali stress than the single-strain treatment and equal-proportion treatment.

**TABLE 1:** Statistics of wheat root growth scanning indexes under different treatments.

Application group	Total length/cm	Total root surface area/cm^2^	Root total volume/cm^3^	Connection number	Apical number
CK	100.12 ± 8.81	6.91 ± 0.90	0.06 + 0.04	824.00 ± 173.00	167.00 ± 9.00
5-33	173.53 ± 75.86	14.20 ± 7.60	0.15 ± 0.03	1790.00 ± 290.00	334.00 ± 70.00
BJYX	249.21 ± 17.56	21.40 ± 3.80	0.19 ± 0.11	2105.00 ± 259.00	449.00 ± 0.51.00
G51-1	218.17 ± 38.29	19.90 ± 1.80	0.32 ± 0.06	1886.00 ± 422.67	497.00 ± 84.00
S-3	199.28 ± 36.69	14.40 ± 8.30	0.12 ± 0.02	1947.00 ± 478.61	330.00 ± 13.00
MB	262.76 ± 56.58	32.40 ± 1.60	0.51 ± 0.11	2617.00 ± 141.00	297.00 ± 52.00
MBE	200.51 ± 14.09	16.30 ± 2.40	0.41 ± 0.12	1914.00 ± 478.00	452.00 ± 13.00

**FIGURE 5:**
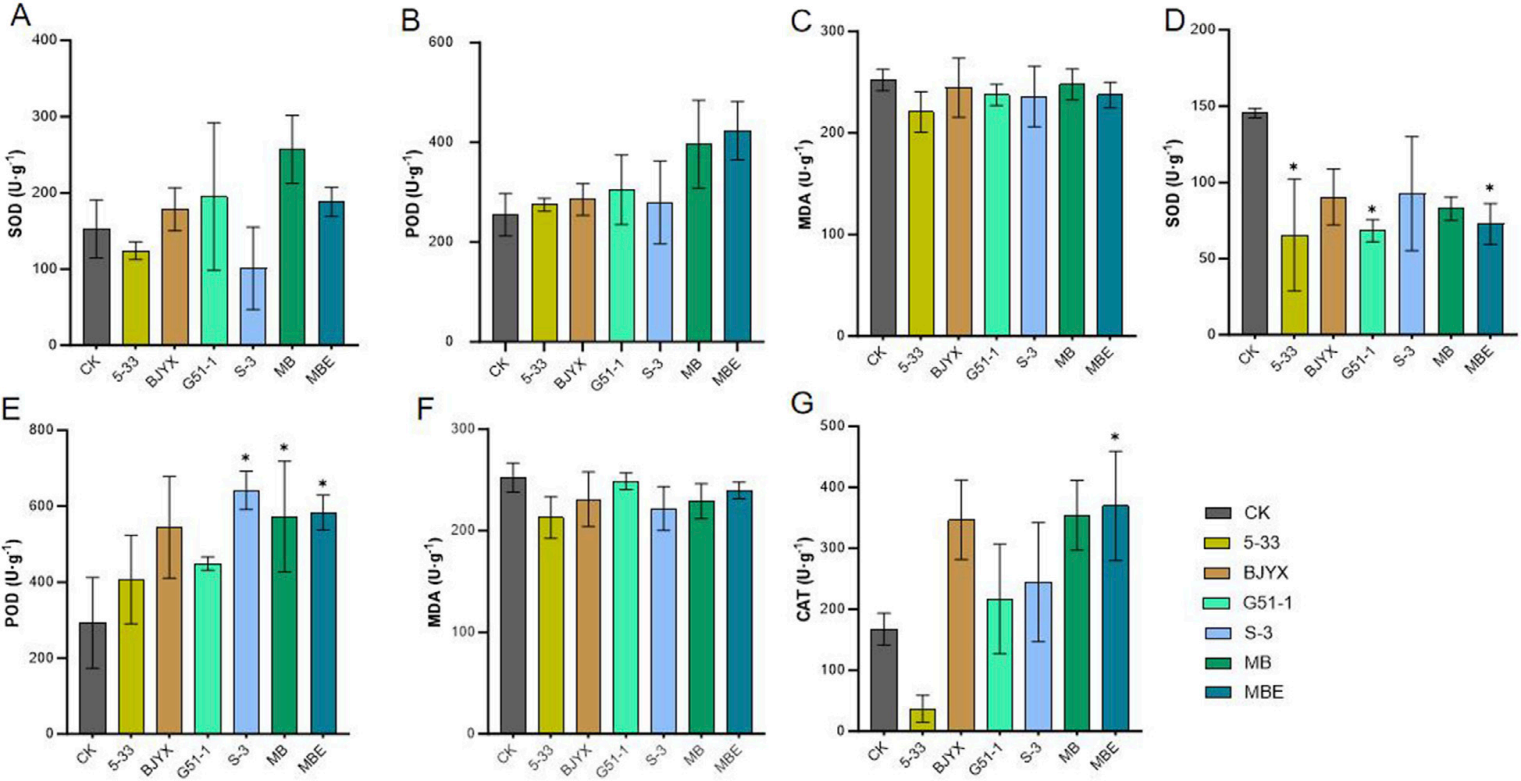
Determination of enzyme activities in wheat leaves and roots treated with MBE, MB, 5-33, BJYX, G51-1, and S-3. The wheat leaves that were subjected to different treatments were selected and ground to determine SOD enzyme activity **(A)**, POD enzyme activity **(B)**, and the concentration of MDA **(C)**. After cleaning, wheat roots were ground and filtered to determine the corresponding root SOD enzyme activity **(D)**, POD enzyme activity **(E)**, MDA concentration **(F)**, and CAT enzyme activity **(G)**. “MB” represents the self-selected mixed bacteria, and “MBE” represents the mixed bacteria based on an equal proportion. Each group was subjected to 3–5 biological treatments. The averages of the data are presented. Error bars represent the standard deviations of the averages. *: *p* < 0.05, significant; **: *p* < 0.01, highly significant.

The enzyme activities of the leaves and roots of wheat seedlings were further determined (Figure 5). In the wheat leaves, compared with those in the control group, the activities of SOD in the BJYX, G51–1, MB, and MBE treatment groups were increased by 16.99, 27.85, 68.38, and 23.34%, respectively (Figure 5A). Peroxidase (POD) activities in the leaves of the 5-33, BJYX, G51-1, S-3, MB, and MBE groups were increased by 7.87, 11.96, 19.73, 9.61, 55.42, and 66.14%, respectively (Figure 5B). The concentrations of MDA in the leaves of the 5-33, BJYX, G51-1, S-3, MB, and MBE treatment groups were reduced by 12.48, 3.01, 5.83, 6.43, 1.74, and 5.85%, respectively (Figure 5C). In the root systems of wheat, compared with those in the control group, the activities of SOD in the 5-33, BJYX, G51-1, S-3, MB, and MBE treatment groups were decreased by 55.02% (*p* < 0.05), 37.85%, 53.03% (*p* < 0.05), 36.31%, 43.03%, and 49.95% (*p* < 0.05), respectively (Figure 5D), whereas POD activities were increased by 38.95%, 86.05%, 53.30%, 119.25% (*p* < 0.05), 95.79% (*p* < 0.05), and 99.32% (*p* < 0.05), respectively (Figure 5E). The concentrations of MDA in the roots of the 5-33, BJYX, G51-1, S-3, MB, and MBE treatment groups were decreased by 15.60, 8.51, 1.42, 12.05, 9.22, and 4.96%, respectively (Figure 5F). Moreover, root peroxidase activities in the BJYX, G51-1, S-3, MB, and MBE treatment groups were increased by 107.14, 29.59, 46.05, 111.51, 120.84% (*p* < 0.05), respectively (Figure 5G).

**FIGURE 6:**
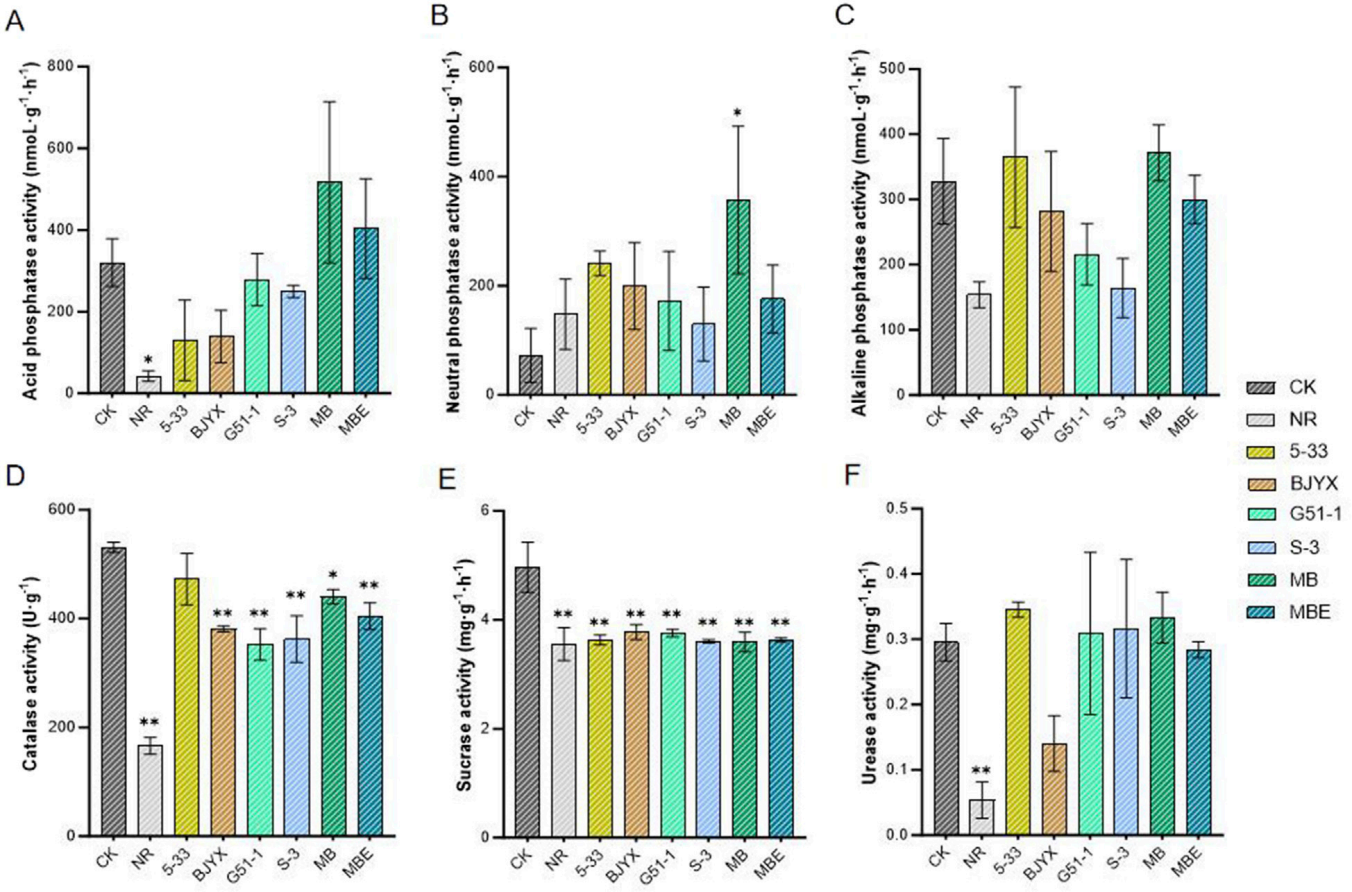
Determination of enzyme activities in the saline-alkaline soil during wheat seedling growth. The activities of acid phosphatase **(A)**, alkaline phosphatase **(B)**, neutral phosphatase **(C)**, peroxidase **(D)**, sucrase **(E)**, and urease **(F)** in the rhizosphere soil of wheat seedlings after 21 days of growth under different treatment conditions and those in the non-rhizosphere soil not treated with the strains were determined. “MB” represents the self-selected mixed bacteria, and “MBE” represents the mixed bacteria based on an equal proportion. “NR” represents the non-rhizosphere soil for “CK”. Each group was subjected to 3–5 biological treatments. The averages of the data are presented. Error bars represent the standard deviations of the averages. *: *p* < 0.05 significant; **: *p* < 0.01 highly significant.

The activities of acid phosphatase, neutral phosphatase, and alkaline phosphatase in the rhizosphere and non-rhizosphere soils of different groups were also determined (Figure 6). The rhizosphere soils generally had higher levels of all three phosphatase enzymes than the non-rhizosphere soils. Compared with those in the control group, the activities of acid phosphatase in the rhizosphere soil of 5–33-, BJYX-, G51-1-, and S-3-treated groups were decreased by 59.43, 56.37, 12.90, and 22.10%, respectively (Figure 6A). Meanwhile, the rhizospheric acid phosphatase activities in the MB and MBE treatment groups were increased by 61.39% and 25.96%, respectively, compared with those in the control group (Figure 6A). The neutral phosphatase activities in the rhizosphere soils of the 5-33, BJYX, G51-1, S-3, MB, and MBE treatment groups were increased by 233.42%, 176.13%, 138.19%, 79.57%, 393.90% (*p* < 0.1), and 142.97%, respectively (Figure 6B). However, the root alkaline phosphatase activities in the BJYX, G51-1, S-3, and MBE groups were decreased by 14.12, 34.15, 49.94, and 8.55%, respectively (Figure 6C). Meanwhile, those in the MB and 5-33 treatment groups were increased by 13.24% and 11.15%, respectively.

In the saline-alkali stress environment, the effects of microbial agents on the activities of soil catalase, sucrase, and urease were simultaneously determined (Figure 6). The rhizosphere soil had higher levels of catalase, sucrase, and urease activities than the non-rhizosphere soil. The catalase activities in the 5-33, BJYX, G51-1, S-3, MB, and MBE treatment groups were all decreased, by 11.06%, 28.27% (*p* < 0.01), 33.71% (*p* < 0.01), 31.82% (*p* < 0.01), 17.15% (*p* < 0.05), and 23.89% (*p* < 0.01), respectively (Figure 6D). Meanwhile, cellulase activities in the 5-33, BJYX, G51-1, S-3, MB, and MBE treatment groups were decreased by 26.83% (*p* < 0.01), 23.88% (*p* < 0.01), 24.30% (*p* < 0.01), 27.36% (*p* < 0.01), 27.56% (*p* < 0.01), and 26.85% (*p* < 0.01), respectively (Figure 6E). Further, the 5-33, G51-1, S-3, and MB treatment groups exhibited increased urease activities by 16.91, 4.54, 7.05, and 12.66%, respectively. Compared to those in the control group, urease activities in the BJYX and MBE treatment groups were decreased by 52.49% and 3.92%, respectively (Figure 6F).

Besides, the application of microbial agents could improve the germination indicators of wheat seeds under saline-alkali stress to varying degrees (Supplementary Table S1). The germination potentials (GP) in the treatment groups of 5–33, G51-1, S-3, MB, and MBE were increased by 6.66, 4.44, 6.66, 10.00, and 10.00%, respectively. The increase of the germination index indicated that the application of microbial agents significantly alleviated the inhibitory effect of saline-alkali stress on the germination of wheat seeds.

## Discussion

Wheat is a major staple crop worldwide, and climate and land changes greatly affect its quality and yield (Qiao et al., 2022). The soil salinization of cultivated land is one important environmental factor that limits wheat production (Yuan et al., 2022). Saline-alkali soil accumulates excessive salt, leading to high osmotic pressure and ion toxicity, inhibiting photosynthesis in wheat plants. In addition, soil compaction caused by salinization restricts nutrient absorption, metabolism, and root growth, thereby hindering the growth and development of wheat plants (Garcia-Lemos et al., 2020; Yuan et al., 2016). Therefore, the management of saline-alkali land is not only important for alleviating negative environmental effects but is also crucial for ensuring the long-term sustainable development of wheat agriculture (Gao et al., 2016). Currently, the most commonly used method for the reclamation of saline-alkali land for wheat growth is drip irrigation scheduling, and multi-level drip irrigation is used to establish vegetation in saline-alkali soil (Dong Q. et al., 2022). However, this requires a long-term comprehensive understanding of the water and salt dynamics under drip irrigation, which requires much work and consumes a large amount of resources.

The development of microbial agents has good prospects for the restoration of saline-alkali land. Applying the JC-K3 bacterial agent increases the biomass accumulation of wheat seedlings under salt stress and fungal community abundances in the rhizosphere soil of wheat seedlings (Ji et al., 2021). In this study, new strains were screened and identified from saline-alkali land, and three of them (*R. aquimaris* S-3, *B. velezensis* G51-1, and *B. subtilis* BJYX) were found to effectively colonize the wheat rhizosphere soil. *Bacillus* species have been defined as beneficial biocontrol bacteria that can promote plant growth with a certain salt tolerance level (Wang et al., 2020; Zhou et al., 2023). For example, a newly isolated strain, *B. subtilis* YB-15, significantly inhibits *Fusarium* crown rot and promotes the growth of wheat seedlings (Xu et al., 2022); Moreover, Ali et al. isolated a salt-tolerant *Bacillus* NMTD17 strain from the Qinghai-Tibet Plateau and showed that this strain has potential to alleviate the effect of saline-alkali soil on crops and reshape rhizosphere bacterial communities (Ali et al., 2022).

To overcome the limitations of saline-alkali land for wheat production, finding new sustainable types of high-affinity specific compound microbial agents is urgent to address the current problem. Secretion by plant roots can provide nutrients for beneficial microorganisms. According to the different types of plant secretions, the enriched microbial communities also vary (Ding et al., 2021). Accordingly, plant self-selection comprises the autonomous selection of suitable microbial communities based on the needs of plant roots to find the optimal microbial treatment group and increase plant yields. In this study, the *in situ* self-selection method was used for wheat plants in saline-alkali soil, and the self-selection *K. dechangensis* 5–33:*R. aquimaris* S-3:*B. subtilis* BJYX:*B. velezensis* G51-1 ratio, based on root zone metagenomic sequencing technology, was determined to be 275:63:5:1 (Figure 3). By adding the composite microbial agent based on this self-selection, the physiological plant height, aboveground and underground fresh weights, and aboveground and underground dry weights of wheat seedlings at 21 days of growth were significantly increased (Figure 4), and each strain was found to promote the growth of wheat plants to varying degrees. This novel combination of microbial agents associated with wheat has good environmental applicability, results in the formation of effective microbial colonies, and exhibits interactions with the environment, showing great potential for the bioremediation of saline-alkali soils.

Soil enzyme activity is a biological indicator of soil ecological functions, is easily influenced by the biological properties of the soil and is an important indicator of changes in soil microbial activity (Dong Shi et al., 2022; Zhen et al., 2019). The application of microbial agents can affect the activities of soil enzymes, the pH, microbial biomass, and other parameters, thereby influencing the root systems of plants and promoting the growth of wheat plants (Duan et al., 2021; Wang and Jiao, 2022). This study demonstrated that applying the composite bacterial agent, based on self-selection, during the growth of wheat plants can increase the activities of neutral phosphatase and urease in the rhizosphere saline-alkali soil to activate soil nutrients while reducing catalase and sucrase activities (Figure 6). Moreover, the addition of this high-affinity composite bacterial agent could significantly improve the physicochemical properties and soil enzyme activities of the wheat rhizosphere in saline-alkali soil. Specifically, POD activity in wheat leaves and roots treated with the compound microbial agent was increased, whereas the MDA content was decreased. The SOD activity in the leaves and the CAT activity in the wheat roots also showed significant increases (Figure 5). The increase in the POD, SOD, and CAT activities would effectively reduce the stress of the saline-alkali soil on wheat plants, especially alleviating damage to their root systems (Figure 4G). The decrease in the MDA content would also alleviate this stress.

**FIGURE 7:**
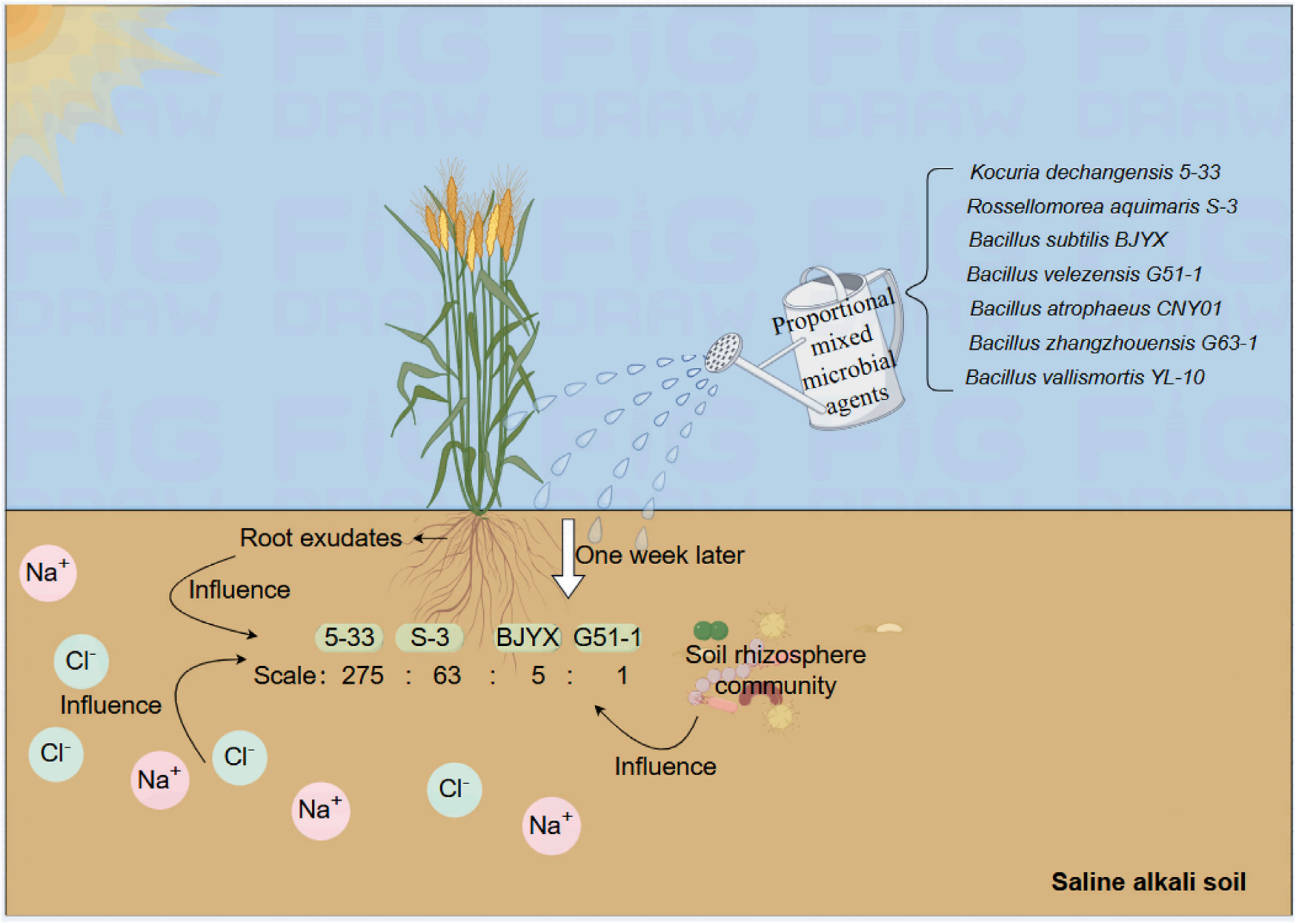
Schematic diagram of the construction of a new type of microbial agent combination (MB) for wheat seedlings. The added and finally selected strains are noted.

The rhizosphere microenvironment is the critical area of interactions among plants, the soil, and microorganisms (Ajilogba et al., 2022). Wheat plants can communicate with soil microorganisms through root exudates (Usyskin-Tonne et al., 2021), triggering a series of biochemical reactions, and this process helps to improve the soil structure and environment through the selection of advantageous microbial communities, ultimately promoting the growth and development of wheat plants. As reported, there are various interactions between saline-tolerant microorganisms and wheat seedlings under multi-factor stress, which affect the metabolism and absorption of wheat seedlings; Meanwhile, wheat seedlings tend to invest more in rhizosphere microorganisms and receive more benefits from them under adverse than favorable soil conditions (Aghili et al., 2014; Zhang et al., 2024). In our study, the composite microbial agent, selected *in situ* from the saline-alkali soil of wheat seedlings, was precisely chosen during the growth and development of wheat seedlings, and its combined activity had a positive and stable promoting effect (Figure 7). By identifying strains through self-selection using wheat seedlings, it is possible to further explore the strains that can help maintain the beneficial growth of wheat seedlings, providing a solid foundation for the screening of beneficial rhizosphere-promoting strains.

In summary, in this study, multiple saline-alkali-tolerant and plant-growth-promoting rhizobacterial strains were screened from raw saline-alkali soil. Through *in situ* selection experiments using wheat seedlings in saline-alkali soil and soil metagenomic analyses, a specialized and high-affinity bacterial agent (MB), suitable for wheat growth under saline-alkali stress, was developed. This study provides microbial resources and a theoretical basis to promote plant growth and development and alleviate saline-alkali stress on plants. This study also suggests a sustainable approach for the improvement and utilization of saline-alkali soil environments.

## Data Availability

The datasets presented in this study can be found in online repositories. The names of the repository/repositories and accession number(s) can be found in the article/Supplementary Material.

## References

[B1] Aghili F. Jan J. Amir H. K. Majid A. Rainer S. Emmanuel F. (2014). Wheat plants invest more in mycorrhizae and receive more benefits from them under adverse than favorable soil conditions. Appl. Soil Ecol. 84, 93–111. 10.1016/j.apsoil.2014.06.013

[B2] Ajilogba C. F. Olanrewaju O. S. Babalola O. O. (2022). Plant growth stage drives the temporal and spatial dynamics of the bacterial microbiome in the rhizosphere of vigna subterranea. Front. Microbiol. 13, 825377. 10.3389/fmicb.2022.825377 35250941 PMC8891599

[B3] Ali Q. Ayaz M. Mu G. Hussain A. Yuanyuan Q. Yu C. (2022). Revealing plant growth-promoting mechanisms of Bacillus strains in elevating rice growth and its interaction with salt stress. Front. Plant Sci. 13, 994902. 10.3389/fpls.2022.994902 36119605 PMC9479341

[B4] Arshad M. J. Khan M. I. Ali M. H. Farooq Q. Hussain M. I. Seleiman M. F. (2024). Enhanced wheat productivity in saline soil through the combined application of poultry manure and beneficial microbes. Bmc Plant Biol. 24, 423. 10.1186/s12870-024-05137-x 38760709 PMC11102207

[B5] Begum N. Hasanuzzaman M. Li Y. Akhtar K. Zhang C. Zhao T. (2022). Seed germination behavior, growth, physiology and antioxidant metabolism of four contrasting cultivars under combined drought and salinity in soybean. Antioxidants (basel) 11 (3), 498. 10.3390/antiox11030498 35326148 PMC8944481

[B6] Chen S. Zhou Y. Chen Y. Gu J. (2018). Fastp: an ultra-fast all-in-one FASTQ preprocessor. Bioinformatics 34 (17), i884–i890. 10.1093/bioinformatics/bty560 30423086 PMC6129281

[B7] Deng Z. Jiang Y. Chen K. Gao F. Liu X. (2020). Petroleum depletion property and microbial community shift after bioremediation using *Bacillus halotolerans* T-04 and *Bacillus cereus* 1-1. Front. Microbiol. 11, 353. 10.3389/fmicb.2020.00353 32194536 PMC7066087

[B8] Derbyshire M. C. Batley J. Edwards D. (2022). Use of multiple ‘omics techniques to accelerate the breeding of abiotic stress tolerant crops. Curr. Plant Biol. 32, 100262. 10.1016/j.cpb.2022.100262

[B9] Ding Y. Yi Z. Fang Y. He S. Li Y. He K. (2021). Multi-omics reveal the efficient phosphate-solubilizing mechanism of bacteria on rocky soil. Front. Microbiol. 12, 761972. 10.3389/fmicb.2021.761972 34956124 PMC8696128

[B10] Dong Q. Zhao X. Zhou D. Liu Z. Shi X. Yuan Y. (2022a). Maize and peanut intercropping improves the nitrogen accumulation and yield per plant of maize by promoting the secretion of flavonoids and abundance of Bradyrhizobium in rhizosphere. Front. Plant Sci. 13, 957336. 10.3389/fpls.2022.957336 35991432 PMC9386453

[B11] Dong S. Wang G. Kang Y. Ma Q. Wan S. (2022b). Soil water and salinity dynamics under the improved drip-irrigation scheduling for ecological restoration in the saline area of Yellow River basin. Agr Water Manage 264, 107255. 10.1016/j.agwat.2021.107255

[B12] Duan C. Mei Y. Wang Q. Wang Y. Li Q. Hong M. (2021). Rhizobium inoculation enhances the resistance of alfalfa and microbial characteristics in copper-contaminated soil. Front. Microbiol. 12, 781831. 10.3389/fmicb.2021.781831 35095795 PMC8791600

[B13] Duan H. Liu W. Zhou L. Han B. Huo S. El-Sheekh M. (2023). Improving saline-alkali soil and promoting wheat growth by co-applying potassium-solubilizing bacteria and cyanobacteria produced from brewery wastewater. Front. Env. Sci-Switz 11, 1170734. 10.3389/fenvs.2023.1170734

[B14] Elliott A. J. Daniell T. J. Cameron D. D. Field K. J. (2021). A commercial arbuscular mycorrhizal inoculum increases root colonization across wheat cultivars but does not increase assimilation of mycorrhiza-acquired nutrients. Plants People Planet 3, 588–599. 10.1002/ppp3.10094 34853824 PMC8607474

[B15] Gao H. Liu J. Eneji A. E. Han L. Tan L. (2016). Using modified remote sensing imagery to interpret changes in cultivated land under saline-alkali conditions. Sustainability-Basel 8, 619. 10.3390/su8070619

[B16] Garcia-Lemos A. M. Großkinsky D. K. Akhtar S. S. Nicolaisen M. H. Roitsch T. Nybroe O. (2020). Identification of root-associated bacteria that influence plant physiology, increase seed germination, or promote growth of the christmas tree species Abies nordmanniana. Front. Microbiol. 11, 566613. 10.3389/fmicb.2020.566613 33281762 PMC7705201

[B17] Hou Y. Zeng W. Hou M. Wang Z. Luo Y. Lei G. (2021). Responses of the soil microbial community to salinity stress in maize fields. Biol. (Basel). 10 (11), 1114. 10.3390/biology10111114 PMC8614889 34827107

[B18] Huson D. H. Beier S. Flade I. Górska A. El-Hadidi M. Mitra S. (2016). MEGAN community edition - interactive exploration and analysis of large-scale microbiome sequencing data. Plos Comput. Biol. 12 (6), e1004957. 10.1371/journal.pcbi.1004957 27327495 PMC4915700

[B19] Ji C. Wang X. Song X. Zhou Q. Li C. Chen Z. (2021). Effect of *Bacillus velezensis* JC-K3 on endophytic bacterial and fungal diversity in wheat under salt stress. Front. Microbiol. 12, 802054. 10.3389/fmicb.2021.802054 34987493 PMC8722765

[B20] Ji C. Wang X. Tian H. Hao L. Wang C. Zhou Y. (2020). Effects of *Bacillus methylotrophicus* M4‐1 on physiological and biochemical traits of wheat under salinity stress. J. Appl. Microbiol. 129, 695–711. 10.1111/jam.14644 32215987

[B21] Jin T. Sun Y. Zhao R. Shan Z. Gai J. Li Y. (2019). Overexpression of peroxidase gene GsPRX9 confers salt tolerance in soybean. Int. J. Mol. Sci. 20, 3745. 10.3390/ijms20153745 31370221 PMC6695911

[B22] Kumawat K. C. Sharma B. Nagpal S. Kumar A. Tiwari S. Nair R. M. (2023). Plant growth-promoting rhizobacteria: salt stress alleviators to improve crop productivity for sustainable agriculture development. Front. Plant Sci. 13, 1101862. 10.3389/fpls.2022.1101862 36714780 PMC9878403

[B23] Li C. L. Tsuang Y. H. Tsai T. H. (2019). Neuroprotective effect of schisandra chinensis on methyl-4-phenyl-1,2,3,6-tetrahydropyridine-induced parkinsonian syndrome in C57BL/6 mice. Nutrients 11 (7), 1671. 10.3390/nu11071671 31330885 PMC6683275

[B24] Li Z. Geng W. Tan M. Ling Y. Zhang Y. Zhang L. (2022). Differential responses to salt stress in four white clover genotypes associated with root growth, endogenous polyamines metabolism, and sodium/potassium accumulation and transport. Front. Plant Sci. 13, 896436. 10.3389/fpls.2022.896436 35720567 PMC9201400

[B25] Martin M. (2011). Cutadapt removes adapter sequences from high-throughput sequencing reads. EMBnet.J. 17, 10–12. 10.14806/EJ.17.1.200

[B26] Menzel P. Kim L. Anders K. (2016). Fast and sensitive taxonomic classification for metagenomics with Kaiju. Nat. Commun. 7, 11257. 10.1038/ncomms11257 27071849 PMC4833860

[B27] Mukherjee P. Mitra A. Roy M. (2019). Halomonas rhizobacteria of avicennia marina of indian sundarbans promote rice growth under saline and heavy metal stresses through exopolysaccharide production. Front. Microbiol. 10, 1207. 10.3389/fmicb.2019.01207 31191507 PMC6549542

[B28] Pervaiz T. Liu S. W. Uddin S. Amjid M. W. Niu S. H. Wu H. X. (2021). The transcriptional landscape and hub genes associated with physiological responses to drought stress in *Pinus tabuliformis* . Int. J. Mol. Sci. 22 (17), 9604. 10.3390/ijms22179604 34502511 PMC8431770

[B29] Qiao Y. Li D. Qiao W. Li Y. Yang H. Liu W. (2022). Development and application of a relative soil water content - transpiration efficiency curve for screening high water use efficiency wheat cultivars. Front. Plant Sci. 13, 967210. 10.3389/fpls.2022.967210 36092403 PMC9459229

[B30] Rahma A. A. Somowiyarjo S. Joko T. (2020). Induced disease resistance and promotion of shallot growth by *Bacillus velezensis* B-27. Pak. J. Biol. Sci. 23 (9), 1113–1121. 10.3923/pjbs.2020.1113.1121 32981242

[B31] Ren H. Zhang F. Zhu X. Lamlom S. F. Zhao K. Zhang B. (2023). Manipulating rhizosphere microorganisms to improve crop yield in saline-alkali soil: a study on soybean growth and development. Front. Microbiol. 14, 1233351. 10.3389/fmicb.2023.1233351 37799597 PMC10548211

[B32] Sahu P. K. Singh S. Singh U. B. Chakdar H. Sharma P. K. Sarma B. K. (2021). Inter-genera colonization of ocimum tenuiflorum endophytes in tomato and their complementary effects on na(+)/k(+) balance, oxidative stress regulation, and root architecture under elevated soil salinity. Front. Microbiol. 12, 744733. 10.3389/fmicb.2021.744733 34733259 PMC8558678

[B33] Sharma S. Kulkarni J. Jha B. (2016). Halotolerant rhizobacteria promote growth and enhance salinity tolerance in peanut. Front. Microbiol. 7, 1600. 10.3389/fmicb.2016.01600 27790198 PMC5062030

[B34] Shi H. Lu L. Ye J. Shi L. (2022). Effects of two *Bacillus velezensis* microbial inoculants on the growth and rhizosphere soil environment of *Prunus davidiana* . Int. J. Mol. Sci. 23 (21), 13639. 10.3390/ijms232113639 36362427 PMC9657632

[B35] Song X. Huang L. Li Y. Zhao C. Tao B. Zhang W. (2022). Characteristics of soil fungal communities in soybean rotations. Front. Plant Sci. 13, 926731. 10.3389/fpls.2022.926731 35812925 PMC9260669

[B36] Sun Y. Wang C. Wang X. Du B. Liu K. Wang C. (2024). Biocontrol characteristics of *Bacillus atrophaeus* CNY01 and its salt-resistant and growth-promoting effect on maize seedling. Biotechnol. Bull. 40, 248. 10.13560/j.cnki.biotech.bull.1985.2023-1174

[B37] Usyskin-Tonne A. Hadar Y. Minz D. (2021). Spike formation is a turning point determining wheat root microbiome abundance, structures and functions. Int. J. Mol. Sci. 22 (21), 11948. 10.3390/ijms222111948 34769377 PMC8585012

[B38] Wang C. Jiao R. (2022). Adaptive pathways of microorganisms to cope with the shift from p-to n-limitation in subtropical plantations. Front. Microbiol. 13, 870667. 10.3389/fmicb.2022.870667 35572659 PMC9100944

[B39] Wang C. Pei J. Li H. Zhu X. Zhang Y. Wang Y. (2024). Mechanisms on salt tolerant of *Paenibacillus polymyxa* SC2 and its growth-promoting effects on maize seedlings under saline conditions. Microbiol. Res. 282, 127639. 10.1016/j.micres.2024.127639 38354626

[B40] Wang C. Zhao D. Qi G. Mao Z. Hu X. Du B. (2020). Effects of *Bacillus velezensis* FKM10 for promoting the growth of *Malus hupehensis* rehd. and inhibiting *Fusarium verticillioides* . Front. Microbiol. 10, 2889. 10.3389/fmicb.2019.02889 31998247 PMC6965166

[B41] Wang J. Lv P. Yan D. Zhang Z. Xu X. Wang T. (2022a). Exogenous melatonin improves seed germination of wheat (*Triticum aestivum* L.) under salt stress. Int. J. Mol. Sci. 23 (15), 8436. 10.3390/ijms23158436 35955571 PMC9368970

[B42] Wang K. Zhang L. Liu Y. Pan Y. Meng L. Xu T. (2015). *Kocuria dechangensis* sp. nov., an actinobacterium isolated from saline and alkaline soils. Int. J. Syst. Evol. Micr 65, 3024–3030. 10.1099/ijs.0.000372 26048314

[B43] Wang K. Zhang N. Fu X. Zhang H. Liu S. Pu X. (2022b). StTCP15 regulates potato tuber sprouting by modulating the dynamic balance between abscisic acid and gibberellic acid. Front. Plant Sci. 13, 1009552. 10.3389/fpls.2022.1009552 36186016 PMC9523429

[B44] Xiao J. Guo X. Qiao X. Zhang X. Chen X. Zhang D. (2021). Activity of fengycin and iturin a isolated from *Bacillus subtilis* Z-14 on *Gaeumannomyces graminis* var. tritici and soil microbial diversity. Front. Microbiol. 12, 682437. 10.3389/fmicb.2021.682437 34220767 PMC8250863

[B45] Xiao X. Wang J. Li J. Li X. Dai X. Shen R. (2022). Distinct patterns of rhizosphere microbiota associated with rice genotypes differing in aluminum tolerance in an acid sulfate soil. Front. Microbiol. 13, 933722. 10.3389/fmicb.2022.933722 35783428 PMC9247542

[B46] Xu W. Yang Q. Xie X. Goodwin P. Deng X. Zhang J. (2022). Genomic and phenotypic insights into the potential of *Bacillus subtilis* YB-15 isolated from rhizosphere to biocontrol against crown rot and promote growth of wheat. Biol. (Basel). 11 (5), 778. 10.3390/biology11050778 PMC9138608 35625506

[B47] Ye S. Yan R. Li X. Lin Y. Yang Z. Ma Y. (2022). Biocontrol potential of *Pseudomonas rhodesiae* GC-7 against the root-knot nematode *Meloidogyne graminicola* through both antagonistic effects and induced plant resistance. Front. Microbiol. 13, 1025727. 10.3389/fmicb.2022.1025727 36386722 PMC9651087

[B48] Yuan G. Liu J. An G. Li W. Si W. Sun D. (2021). Genome-wide identification and characterization of the trehalose-6-phosphate synthetase (TPS) gene family in watermelon (*Citrullus lanatus*) and their transcriptional responses to salt stress. Int. J. Mol. Sci. 23 (1), 276. 10.3390/ijms23010276 35008702 PMC8745194

[B49] Yuan G. Sun D. An G. Li W. Si W. Liu J. (2022). Transcriptomic and metabolomic analysis of the effects of exogenous trehalose on salt tolerance in watermelon (*Citrullus lanatus*). Cells-Basel 11 (15), 2338. 10.3390/cells11152338 PMC9367363 35954182

[B50] Yuan Y. Zhong M. Shu S. Du N. Sun J. Guo S. (2016). Proteomic and physiological analyses reveal putrescine responses in roots of cucumber stressed by NaCl. Front. Plant Sci. 7, 1035. 10.3389/fpls.2016.01035 27471514 PMC4945654

[B51] Zhang G. Bai J. Zhai Y. Jia J. Zhao Q. Wang X. (2024). Microbial diversity and functions in saline soils: a review from a biogeochemical perspective. J. Adv. Res. 59, 129–140. 10.1016/j.jare.2023.06.015 37392974 PMC11081963

[B52] Zhang S. Kong J. Chen L. Guo K. Zhou X. (2022a). Increased tea saponin content influences the diversity and function of plantation soil microbiomes. Microbiol. Spectr. 10 (1), e0232421. 10.1128/spectrum.02324-21 35019691 PMC8754145

[B53] Zhang X. Hua Y. Liu Y. He M. Ju Z. Dai X. (2022b). Wide belt sowing improves the grain yield of bread wheat by maintaining grain weight at the backdrop of increases in spike number. Front. Plant Sci. 13, 992772. 10.3389/fpls.2022.992772 36061798 PMC9433909

[B54] Zhao D. Ding Y. Cui Y. Zhang Y. Liu K. Yao L. (2022). Isolation and genome sequence of a novel phosphate-solubilizing rhizobacterium *Bacillus altitudinis* GQYP101 and its effects on rhizosphere microbial community structure and functional traits of corn seedling. Curr. Microbiol. 79 (9), 249. 10.1007/s00284-022-02944-z 35834051

[B55] Zhen Z. Wang S. Luo S. Ren L. Liang Y. Yang R. (2019). Significant impacts of both total amount and availability of heavy metals on the functions and assembly of soil microbial communities in different land use patterns. Front. Microbiol. 10, 2293. 10.3389/fmicb.2019.02293 31636621 PMC6788306

[B56] Zhou B. Jia R. Chen X. Yang L. Duan M. Xiao F. (2023). Impact of bacteria-nitrogen coupling on cotton growth and nitrogen utilization under different salt stress. Agr Water Manage 280, 108221. 10.1016/j.agwat.2023.108221

