# Supplementary material for: Growth-promoting effects of self-selected microbial community on wheat seedlings in saline-alkali soil environments
Source: Front Bioeng Biotechnol. 2024 Dec 13;12:1464195. doi: 10.3389/fbioe.2024.1464195 (PMC11671506; doi:10.3389/fbioe.2024.1464195)
Supplement: Supplementary file 1 [file DataSheet1.docx]

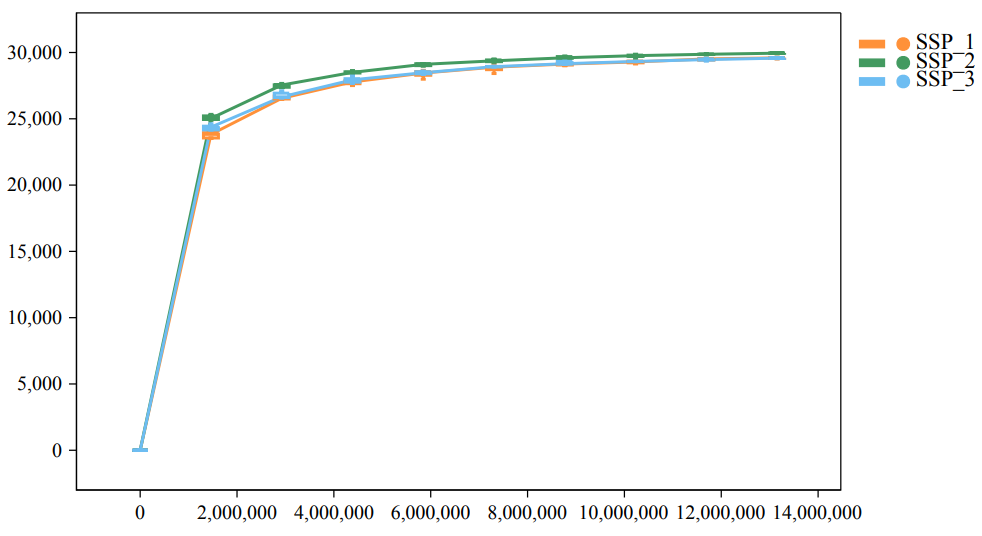


**Supplementary Figure S1** The rarefaction curves of different samples.

.

**Supplementary Table S1** Effects of different strain treatments on seed germination of wheat under saline-alkali stress

| Treatment | GP（%） | GR（%） | GI |
| --- | --- | --- | --- |
| 0+N | 95.56±1.11 | 100.00±0.00 | 42.84±0.25 |
| 1+N | 56.67±2.22 | 70.00±6.66 | 22.45±0.49 |
| 1+5-33 | 63.33±3.33 | 76.67±3.33 | 26.91±0.98 |
| 1+BJYX | 55.56±2.22 | 68.89±1.11 | 23.39±1.23 |
| 1+G51-1 | 61.11±5.56 | 75.56±7.78 | 26.29±0.88 |
| 1+S-3 | 63.33±6.67 | 77.78±7.78 | 26.75±0.87 |
| 1+CNY01 | 51.11±5.56 | 68.88±1.89 | 23.21±0.64 |
| 1+YL-10 | 54.44±1.11 | 71.11±7.78 | 23.22±0.39 |
| 1+G63-1 | 54.44±2.22 | 70.00±3.33 | 23.46±1.01 |
| 1+MB | 66.67±6.67 | 76.66±4.41 | 28.23±0.16 |
| 1+MBE | 66.67±3.33 | 76.66±5.52 | 28.06±0.53 |

Note: “0+” represents the saline-alkali-free condition. “1+” represents the simulated saline-alkali condition. “N” represents no strains added. “MB” represents the self-selected mixed bacteria. “MBE” represents the mixed bacteria of the selected strains based on an equal proportion.
